# Advancing assessment of responsive feeding environments and practices in child care

**DOI:** 10.1017/jns.2024.10

**Published:** 2024-03-07

**Authors:** Julie E. Campbell, Jessie-Lee D. McIsaac, Margaret Young, Elizabeth Dickson, Sarah Caldwell, Rachel Barich, Misty Rossiter

**Affiliations:** 1 Early Childhood Collaborative Research Centre, Mount Saint Vincent University, Halifax, Canada; 2 School of Health and Human Performance, Dalhousie University, Halifax, Canada; 3 Faculty of Education and Department of Child and Youth Study, Mount Saint Vincent University, Halifax, Canada; 4 Department of Applied Human Sciences, University of Prince Edward Island, Charlottetown, Canada

**Keywords:** child care environments, children, coaching, nutrition, responsive feeding

## Abstract

Child care environments offer an ideal setting for feeding interventions. CELEBRATE Feeding is an approach implemented in child care environments in two Maritime Provinces in Canada to support responsive feeding (RF) to foster children’s self-efficacy, self-regulation, and healthy relationships with food. This study aimed to describe RF in child care using established and enhanced scoring frameworks.

The Environment and Policy Assessment and Observation (EPAO) was modified to reflect RF environments and practices, resulting in our modified EPAO and a *CELEBRATE* scale. Observations were conducted in 18 child care rooms. Behaviours and environments were scored on both scales, creating 21 RF scores, with a score of ‘3’ indicating the most responsiveness. Descriptive analyses of the scores were conducted. The overall room averages were Mean (M) = 41.00, Standard Deviation (SD) = 7.07 (EPAO), and M = 37.92 SD = 6.50 (*CELEBRATE*). Most responsive scores among rooms within our EPAO and *CELEBRATE* scales, respectively, were ‘educators not using food to calm or encourage behaviour’ (M = 2.94, SD = 0.24; M = 2.98, SD = 0.06) and ‘not requiring children to sit at the table until finished’ (M = 2.89, SD = 0.47; M = 2.97, SD = 0.12). The least responsive scores within the EPAO were ‘educator prompts for children to drink water’ (M = 0.78, SD = 0.94) and ‘children self-serving’ (M = 0.83, SD = 0.38). The least responsive in the *CELEBRATE* scale were ‘enthusiastic role modelling during mealtime’ (M = 0.70, SD = 0.68) and ‘praise of mealtime behaviour unrelated to food intake’ (M = 0.74, SD = 0.55). The *CELEBRATE* scale captured unique observation information about RF to allow documenting change over time with detailed measurement to inform and support nutrition interventions within child care environments.

## Introduction

The early years of a child’s life are a critical period for learning and brain development offering an ideal timeframe for health interventions.^(1,2)^ The experiences and environments a child encounters during these years help provide the foundation for their lifelong health, learning, and well-being.^(1,3–5)^ More young children are spending time in child care settings;^(6,7)^ as a result, creating health-promoting child care environments is fundamental to supporting the future generation’s long-term success.^(8–10)^ It is well-established that a balanced, varied, and nutrient-dense diet is essential for children’s growth and development to avoid the potential of malnutrition, obesity, and diet-related chronic diseases.^(11–13)^ Since young children consume a large portion of their daily food intake in child care settings, dietary intake is often a focus of public health nutrition in these programmes.^(14)^ However, focusing on food intake alone does not consider the potential influences of the environment and practices in which the food is explored, offered, and consumed on child development and health behaviours.^(12,13,15–18)^ For example, responsive feeding practices, such as not pressuring the child to eat and allowing self-feeding, strengthen children’s self-efficacy, self-regulation, and emotional management and can help children foster healthy eating habits and healthy relationships with food, reducing both child under- and over-nutrition.^(15,19–22)^ Responsive feeding is commonly characterized by prompt and developmentally appropriate responses by caregivers to children’s signals of hunger and satiety,^(19,23)^ along with the context and physical environment in which it occurs.^(23)^ However, while there is a growing emphasis on responsive feeding, caregivers who lack confidence in children’s ability to regulate their intake appropriately, or those who feel stressed about mealtime, may unknowingly engage in less responsive feeding practices.^(19,24)^

Child care programmes across Canada often follow nutrition standards that align with national Canada’s Food Guide recommendations.^(25)^ However, research suggests that feeding environments in child care around the world may not always be responsive, where caregivers may override children’s internal cues of hunger and satiety by controlling food intake, restricting certain foods, or using food as a reward.^(26–28)^ In addition, creating and maintaining responsive feeding practices and environments can also be challenging due to caregivers’ social and cultural beliefs influencing their feeding practices^(29–32)^ as well as caregivers having a lack of time and resources.^(19,32)^ Interventions to support responsive feeding are needed to address the unique barriers to implementation in child care settings.^(33)^

Measurement of responsive feeding practices in child care can help inform public health nutrition interventions, but there are limited instruments that are used to observe and assess all aspects of responsive feeding in child care settings; rather, many of the existing tools are focused within the home environment with parent practices^(34)^ or do not include both environmental and behavioural aspects of responsive feeding, including food-related practices that occur outside of meal time, such as food exploration and food-related play and activities.^(35–37)^ One widely used and internationally recognized public health nutrition tool in child care environments that provides a foundation for these components of responsive feeding is the Environment and Policy Assessment and Observation instrument, 2017 version (EPAO-2017).^(38,39)^ The original EPAO was first designed in the early 2000s to assess the nutrition and physical activity environments, including educators’ responsive feeding interactions and practices, at child care centres using a combination of direct observation and review of programme-based policies and practices, consequently capturing *best practices* in a child care environment.^(38)^ However, while the EPAO has been recognized as a comprehensive and evidence-based tool, the best practices have been built through an emphasis on combatting an obesogenic environment,^(40)^ resulting in a greater valuing toward *eating* healthy foods as desirable behaviour through encouragement and praise.^(15,41)^ Conversely, the recent emphasis on responsive feeding emphasizes building confident and competent eaters to build positive relationships with food for life through providing opportunities for exposure to a variety of foods, without the pressure to eat.^(19,27,42–44)^

The rigorous development, inclusion of the broader feeding environment throughout the full day, and widespread use of the EPAO offers a useful foundation to consider adaptations that reflect responsive feeding practices and environments; however, a more comprehensive tool would help to record a more nuanced assessment of responsive feeding practices and guide public health nutrition interventions. Therefore, the purpose of this paper is to describe responsive feeding practices in child care environments in Nova Scotia and Prince Edward Island (Canada) by advancing established scoring frameworks. The results can inform data collection for interventions in child care, such as those focused on coaching to support responsive feeding.

## Methods

### Research context

Coaching in Early Learning Environments to Build a Responsive Approach to Eating and Feeding (CELEBRATE Feeding) was a behaviour change theory-based feasibility study in Nova Scotia and Prince Edward Island (Canada) that aimed to enhance responsive feeding in child care settings through a coaching intervention based on practices that support responsive feeding environments.^(33)^ The coaching intervention was informed by the Behavior Change Wheel (BCW) framework to guide behaviour change.^(45)^ Within the BCW, intervention development comprises three steps, the first being understanding the behaviour.^(46)^ Through this process, and in the context of the CELEBRATE Feeding project, the coach determined behaviours that may benefit from change using a data collection tool modified from the EPAO-2017. Through this study, the CELEBRATE Feeding approach was developed, which builds on previous definitions of responsive feeding by highlighting the importance of language, play, diversity, inclusion, and celebration in early learning environments (Rossiter MD, Young M, Dickson E, et al., under review). This approach to responsive feeding supports children being exposed to a variety of foods, using various exposure methods, in a predictable, safe, and supportive environment, without the pressure to eat more or less of certain foods.

### Study design and participants

The current study focuses on cross-sectional observation data collected from participating child care programmes at the baseline of the CELEBRATE Feeding project. Nine child care centres (including 18 child care rooms within the centres) from Nova Scotia and Prince Edward Island participated in the current study following a publicly promoted call for interest by email and social media. Exploring responsive feeding in the Maritimes is important, especially in light of the growing policy focus on early years and the recognized imperative to address elevated rates of chronic disease and food insecurity.^(47–49)^ Centres were purposively selected based on interest and capacity to participate in the intervention following an information session, while ensuring a mixture of licensing capacity sizes (maximum 80 children) and demographics from various regions within the provinces. Informed, written consent to participate was gathered from each director, educator, and parent of children being observed.

### Modified tools

#### Our modified EPAO-2017

To measure responsive feeding, a modified EPAO-2017 tool^(38,39)^ was used in accordance with its nature as a flexible tool to best reflect research needs.^(50–52)^ Our modified version of the EPAO in this study was created to align with the CELEBRATE Feeding project’s responsive feeding approach (Rossiter MD, Young M, Dickson E, et al., under review) to inform coaching and data collection for the feasibility study. The modification began through a comprehensive review of the EPAO-2017 tool following a review of the literature^(33)^ in consultation with the broader research team with expertise in child development, nutrition, and dietetics. Pilot testing our modified tool in local child care centres provided important local context and helped inform the tool’s layout to facilitate data collection.

The modification of the EPAO-2017 is fully described in Additional File 1. The main modification involved excluding physical activity components from the EPAO, as they were beyond the project’s scope. The revised EPAO primarily focuses on educator behaviours and the feeding environment, omitting the scoring of specific foods consumed by children. Changes also addressed language differences in responsive feeding practices; for example, the tool avoids categorizing foods as ‘healthy’ or ‘unhealthy’, opting for neutral language. Moreover, language involving ‘encouragement’ or ‘praise for eating’, potentially interpreted as mild pressure, was either removed or adapted. Additionally, although not detailed in this study, qualitative questions were incorporated into the modified EPAO to capture contextual information about responsive feeding environments.

#### CELEBRATE scale

We felt that one of the main limitations of the EPAO-2017 is that it does not consider the frequency or breadth of practices; rather, observation items are only nominally scored as Yes (1) or No (0).^(53)^ Due to the varying contexts of the participating child care centres (e.g. size of room, number of educators/children, observer proximity), to capture differences in the responsive feeding behaviour observational items, it was decided that two 4-point scales would provide more nuanced and detailed observation information to inform future coaching. Thus, in our modified EPAO, the first 4-point scale reflected the proportion of educators doing the behaviour (0 = none, 1 = some, 2 = most, 3 = all), and the second reflected the frequency of the action (0 = none, 1 = some, 2 = most, 3 = all). The average value derived from these two scales has been coined the *CELEBRATE* scale. Rather than simply categorizing the behaviour as occurring or not, this scale allowed the research team to quantify differences between rooms and track progress more closely. However, some observation items were not subject to the breadth of educators and frequency of a behaviour (e.g. environmental observations, serving style, planned nutrition education) and allowed more dichotomous scoring like the EPAO-2017.

### Data collection

Trained research staff conducted day-long observations between July and September 2022 in two rooms at each participating child care centre (n = 18). Separate rooms were observed each day, with observations starting before the morning snack and finishing after the afternoon snack with a small break during the children’s rest time. Two researchers were present for each observation, sat in different areas of the room, and took raw notes on the room environment, meals, activities, and conversations throughout the day. After the observation, researchers independently typed up their raw notes and then completed our modified version of the EPAO, scoring the appropriate items on the *CELEBRATE* scale. Once the modified EPAO scores were completed for individual rooms, the researchers met to review the scores and resolve any discrepancies, agreeing on scores that were inputted into our final modified EPAO document.

### Data analysis

Demographic characteristics of participating centres and individual rooms were obtained and analysed descriptively. Centres’ locations were classified per Statistics Canada’s Population Centre and Rural Area Classification 2016 using their postal code.^(54)^ The scoring and analysis of our modified EPAO occurred in two different ways using SPSS v. 27.0 (IBM Corp, Armonk, USA). First, to include a comparison with EPAO-2017 and its scoring procedures, the obtained *CELEBRATE* scale scores were re-coded to reflect its Yes/No scoring. This was calculated such that any value greater than 0 calculated from the average value of the *CELEBRATE* scale was coded as a ‘Yes’ and an average value of 0 was coded as a ‘No’. From there, 21 relevant Nutrition scores that aligned with our approach (Rossiter MD, Young M, Dickson E, et al., under review) were calculated based on the EPAO-2017 user manual^(39)^ scoring details. Each was scored from 0 to 3 (3 as best practice or most responsive, 0 as not occurring, and all other values ranging between these). Although the EPAO-2017 groups scores into subcomponents, we summed the values of the Nutrition Scores for each observation room to give an overall nutrition score ranging from 0 to 63. This reflected the full scope of the Nutrition Scores included rather than weighting scores within a construct.

The second method of scoring reflects the *CELEBRATE* scale values from the observations. The same 21 nutrition scores variables were calculated; however, instead of using the 0–1 values for the respective observation items, the average value given by the *CELEBRATE* scale for each item involved in the nutrition score was summed and divided by the number of meals and activity periods observed, so that the values would be comparable between rooms that may have had fewer observed meals or activity periods, similar to how the EPAO-2017 manual outlines the scoring.^(39)^ Then, for each score, the highest possible value was determined to form a scale for the score. In other cases, the highest possible value was higher if the nutrition score combined various observation items; thus, the final score was scaled to be out of 3. Any negatively framed nutrition scores were reverse-coded and renamed to continue to reflect a score of 3 as the best practice, also done in the EPAO-2017 manual scoring. Each nutrition score was summed to give an overall *CELEBRATE* scale nutrition score for each room, also out of a possible 63 points. Then, the sum of all nutrition scores for each room was calculated and the average score among all rooms was assessed for both scoring methods. To identify specific areas of strength or weakness of the individual nutrition scores, the mean and standard deviation of each nutrition score across all rooms were analysed.

## Results

Table 1 presents the demographic characteristics of the rooms in each child care centre. Of the nine centres observed, five were in Nova Scotia and four were in Prince Edward Island. The majority (33.3%) of centres were located in small population centres (1,000 – 29,999 people), and almost half (44%) of rooms observed in the centres were of the preschool (3y-5y) age group. The mean licensing capacity of all centres was 50 children, and each room averaged 10.7 children.

**Table 1.** Room demographics

Variable	n	%	
Rooms	18		
Province			
Nova Scotia	10	55.6	
Prince Edward Island	8	44.4	
Location			
Rural (<1,000 persons)	4	22.2	
Small population centre (1,000–29,999 persons)	6	33.3	
Medium population centre (30,000–99,999 persons)	4	22.2	
Large urban population centre (≥100,000 persons)	4	22.2	
Children’s age groups, n (%)			
<18 months	4	22.2	
18 months -<3 years	3	16.7	
3 years-5 years	8	44.4	
Mixed age (18 months-5 years)	3	16.7	
	Mean	SD	Range
Number of children per room, M (SD) (range)	10.7	5.2	2.0–20.0

Table 2 provides both scales’ total nutrition scores per room, meaning that the 21 scores out of 3 points for each room were summed out of a possible 63 points. For our modified EPAO scores, the overall average and standard deviation was 41.00 (7.07). The highest score per room was 50.00 in a small infant room within a rural centre. The lowest overall score was 27.00 in a small infant room within a small population centre location. As for the *CELEBRATE* scale scores, the overall average and standard deviation was lower at 37.92 (6.50), demonstrating the scale’s ability to capture some differences. The highest and lowest scores were from the same two rooms as above, with scores of 47.93 and 28.00, respectively, again showing some differences that could inform coaching.

**Table 2.** Average total nutrition scores per room

Room #	Location	Age Group	# children present		Our modified EPAO scale		*CELEBRATE* scale	
1	Medium pop. centre	3 years-5 years	7		45.00		41.72	
2	Medium pop. centre	3 years-5 years	16		48.00		43.29	
3	Rural	<18 months	3		50.00		47.93	
4	Rural	3 years-5 years	7		47.00		44.63	
5	Medium pop. centre	Mixed age	7		44.00		42.55	
6	Medium pop. centre	Mixed age	16		49.00		44.80	
7	Rural	3 years-5 years	11		38.00		33.61	
8	Rural	3 years-5 years	14		47.00		38.42	
9	Large urban pop. centre	18 months - <3 years	13		40.00		36.30	
10	Large urban pop. centre	3 years-5 years	20		42.00		37.63	
11	Small pop. centre	<18 months	4		27.00		28.00	
12	Small pop. centre	3 years-5 years	12		33.00		30.04	
13	Small pop. centre	<18 months	5		31.00		29.73	
14	Small pop. centre	18 months - <3 years	16		30.00		28.10	
15	Small pop. centre	<18 months	2		42.00		42.88	
16	Small pop. centre	Mixed age	12		48.00		45.02	
17	Large urban pop. centre	18 months - <3 years	14		37.00		31.71	
18	Large urban pop. centre	3 years-5 years	13		40.00		36.26	
			M	SD	M	SD	M	SD
Average			10.67	5.20	41.00	7.07	37.92	6.50

Table 3 identifies each nutrition score with our modified EPAO scale and the *CELEBRATE* scale (0–3 scales, with 3 as best practice or most responsive). The highest achieving nutrition score for our modified EPAO was ‘educators not using food to calm or encourage appropriate behaviour’ (M = 2.94, SD = 0.24). Other areas that centres were achieving close to best practice included ‘educators not requiring children to sit at the table until finished’ (M = 2.89, SD = 0.47), ‘educators sitting with children at mealtime’ (M = 2.78, SD = 0.73), and ‘educators using neutral language when talking about food/feeding’ (M = 2.78, SD = 0.55). The *CELEBRATE* scale column reveals more nuanced scores indicating not just if the item is happening but how often or to what extent the practice is occurring [see Additional File 2 for examples]. The centres performed best on the same top two behaviours as our modified EPAO scoring (M = 2.98, SD = 0.06 and M = 2.97, SD = 0.12, respectively), as well as ‘the educators not using food for bribes/rewards or giving rewards for eating’ (M = 2.93, SD = 0.14).

**Table 3.** Scoring per nutrition score across rooms (n = 18)

Nutrition Scores	Our modified EPAO (0–3)		*CELEBRATE* scale (0–3)	
	Mean	SD	Mean	SD
Educators eat same food as children	1.89	1.28	0.95	0.79
Educators do not eat unhealthy foods	2.61	0.61	2.88	0.10
Educators enthusiastically role model feeding	1.50	1.15	0.70	0.68
Educators sit with children	2.78	0.73	1.88	0.77
Educators do not provide verbal praise related to food intake	1.50	1.10	2.36	0.54
Educators ask if children are hungry before serving seconds	2.44	1.15	1.97	1.10
Educators do not require children to sit at table until finished	2.89	0.47	2.97	0.12
Educators provide gentle comments or nudges towards feeding	2.00	1.08	1.06	0.81
Educators do not use food for food bribes/rewards or give rewards for eating	2.67	0.59	2.93	0.14
Educators do not use food to calm or encourage appropriate behaviour	2.94	0.24	2.98	0.06
Educators provide prompts for children to drink water	0.78	0.94	1.00	1.03
Educators do not force or coerce children to eat	2.28	1.02	2.67	0.47
Educators encourage food exploration	1.78	1.26	1.09	1.02
Educators provide praise for mealtime practices and self-regulation	1.11	1.13	0.74	0.55
Educators use neutral language when talking about food/feeding	2.78	0.55	2.51	0.82
Educators do not pressure children to eat	1.33	1.08	2.20	0.64
Educators provide informal nutrition education (talk about foods they are eating, importance of healthy eating)	1.94	0.42	1.25	0.60
*The below items were not scored using a CELEBRATE scale because the breadth and frequency were not relevant but its value from our modified EPAO is included in CELEBRATE scale summed scores.*				
Drinking water never restricted to children	1.94	1.39		
Children served themselves most/all foods and decided what portion sizes to take	0.83	0.38		
Centre has an active garden	1.76	1.39		
Educators deliver planned nutrition education to children (nutrition, healthy eating, cooking, or meal preparation activities/books)	1.33	1.53		

Some of the lowest scores among all centres within our modified EPAO score included ‘educators providing prompts for children to drink water’ (M = 0.78, SD = 0.94), ‘children serving themselves most/all foods and deciding what portion sizes to take’ (M = 0.83, SD = 0.38), and ‘educators providing praise for mealtime practices and self-regulation’ (M = 1.11, SD = 1.13). The lowest scores for the *CELEBRATE* scale were ‘educators enthusiastically role modelling feeding’ (M = 0.70, SD = 0.68) and ‘educators providing praise for mealtime practices and self-regulation’ (M = 0.74, SD = 0.55).

## Discussion

This paper describes the current state of responsive feeding environments and practices by advancing scoring frameworks in child care centres from a study in Prince Edward Island and Nova Scotia. Day-long observations in two rooms per centre were conducted and analysed using our modified EPAO-2017 and *CELEBRATE* scales. Many responsive feeding practices were being implemented in the participating child care centres. As for the overall room scores, the majority of the rooms scored on the higher half of possible scores (>31.5 (out of 63)), indicating positive responsive feeding environments and practices and an opportunity for enhancements through a coaching intervention. The observations with our modified EPAO and *CELEBRATE* scales found that generally, rooms performed best in responsive feeding behaviours that dictate what *not* to do. For example, when looking at the reported nutrition score means, both scales found that rooms were most responsive in ‘educators not using food to calm or encourage appropriate behaviour’ and ‘educators not requiring children to sit at table until finished’. Whereas some of the behaviours scoring the lowest were ‘educators providing prompts for children to drink water’, ‘children serving themselves most/all foods and deciding what portion sizes to take’, ‘enthusiastic role modelling’, and ‘educators providing praise for mealtime practices and self-regulation’, which are all desirable behaviours. These findings are consistent with another study that used the EPAO to observe nutrition practice-related items of early child care educators, where they also rarely observed undesirable practices they deemed ‘controlling’; thus, indicating greater responsiveness, and those practices identified as desirable or ‘healthful’ were much more scattered and inconsistently observed.^(55)^

The result might be because educators are not aware of beneficial responsive feeding practices for children. Currently the nutrition guidance documents in Nova Scotia^(56)^ and Prince Edward Island^(57)^ are outdated (based on an older version of Canada’s Food Guide) and do not encompass more recent research evidence on responsive feeding. However, even if the guidelines were up-to-date, having knowledge alone does not guarantee behaviour change. Rather than just providing information, Ajzen et al.^(58)^ suggest identifying existing beliefs and their impact on actions, then providing information to challenge, strengthen, or form new beliefs to support the desired behaviour. Moreover, when learning about best practice behaviours related to food and feeding, or information processing and learning in general, people cannot process everything equally. Information gets prioritized based on what is deemed most important, and ‘bad’ or ‘negative’ information tends to draw stronger attention and processing, leading to enhanced memory.^(59)^ Also, when learning to do a task, various studies have found that the motivation to avoid punishment or consequences for doing something wrong is stronger than the motivation of positive reinforcement or incentives.^(59)^ These behavioural principles may be influencing why educators seemed to be better at avoiding the ‘bad’ or undesirable behaviours regarding responsive feeding.

Other contributing factors such as attitudes, beliefs, perceptions of control, and social context may also influence the feeding behaviours of educators in child care settings. A 2017 study used semi-structured interviews to assess child care providers’ perspectives on responsive feeding practices with children ages 2–5 years.^(29)^ The factors that influenced their experiences implementing responsive feeding practices included differing views on if children were capable of self-regulating their food consumption, the belief that portion size guidelines had to be consumed rather than just offered, and limited food availability. Each of these factors affected their abilities to allow children to serve themselves and decide on what and how much to eat.^(29)^

Another reason for the potentially low scores in children serving themselves in the current study stems from the context of the COVID-19 pandemic. Various studies on the impact of COVID-19 on nutrition and feeding practices in early childhood settings have noted that they used to take part in family-style meals, allowing the children to serve themselves; however, the regulations throughout the pandemic did not allow for children to be involved in the handling of food.^(60–62)^ This was also noted anecdotally throughout the conversations and observations at the centres. This may be something that is slowly being reintegrated into feeding practices, but as a result of the longevity of restrictions, these feeding habits and routines may take time, support, and education to be reintroduced. Acknowledging each of these potential underlying factors that may have led to less responsive behaviours, and using our modified tool to gain more context into these behaviours has informed our CELEBRATE Feeding approach, where capability, motivation, and opportunity were all considered in an effort to enable behaviour change through techniques, such as environmental restructuring, education and training, or modelling of behaviours, to influence their future responsive feeding environment and practices.^(45)^

### Importance of the modified tools

Modifying the EPAO-2017 was important as other tools that measure responsive feeding are often aimed at parents, are in self-report format, or do not include the comprehensive nature of both responsive feeding environments and behaviours throughout the whole day.^(34–37)^ Further, it required some modifications to its language for the purpose of the CELEBRATE Feeding approach to best align with current evidence on responsive feeding. The other modification, which was also an issue identified by Byrne et al.,^(53)^ was to expand the way the observation items were scored, such that behaviours were scored for their frequency and breadth using the *CELEBRATE* scale rather than simply happening or not, which was how the EPAO-2017 was scored. This allowed for more detailed observation and scoring, especially given the observations were of multiple educators and various tables of children at meal times. Having this more nuanced score helped inform the coaching intervention to optimally plan for what areas centres could use more support in and to collaboratively set goals with the centres. Another benefit of using the *CELEBRATE* scale is its function in pre-post-intervention comparisons through changes over time. It allows for more subtle changes in behaviour to be documented, beyond just seeing if a behaviour changes from not occurring to occurring after coaching, as that is a limitation of the EPAO-2017.^(51)^ Thus, even small impacts, such as a behaviour occurring ‘some of the time’ to ‘most of the time’ could be detected and the effects of the coaching intervention could be captured more accurately.

### Strengths and limitations

Beyond the benefits of applying the *CELEBRATE* scale, this research displayed various strengths. Beginning with the diversity of centres, the project recruited nine centres across two provinces with differing regulations, population sizes, and licensing capacities. Secondly, observations with trained observers were used to capture the responsive feeding environment and practices, and our modified EPAO tool was implemented as a pilot observation in a separate child care setting before the formal research observations. However, this study is not without limitations. For instance, similar to the EPAO-2017, the observation of each room was completed on only one day. We tried to ensure the observations occurred on a typical day within the room, but this may not have always been the case, with substitute educators sometimes present or an atypical number of children in the room that day. Additionally, our modified EPAO and the *CELEBRATE* scale underwent no formal reliability testing. This was not considered since the primary purpose of the tool was to inform coaching rather than to define a responsive feeding score more broadly. Finally, using the average value of two 4-point scales on the *CELEBRATE* scale, assessing behaviour breadth and frequency, lacks the ability to identify specific facets that may need more attention. This approach may obscure distinctions between scenarios where all educators are partially implementing a behaviour and cases where only a few educators consistently fulfil the behaviour. Thus, future work could consider reporting both parts of the *CELEBRATE* scale to help address this in coaching.

### Implications

The implications of this research extend across various domains, offering valuable insights for both research and practice in the realm of responsive feeding practices in early childhood education. For research, the study highlights the necessity of employing comprehensive tools such as the *CELEBRATE* scale to assess responsive feeding practices thoroughly. This approach not only identifies specific areas of strength and weakness but also allows for nuanced pre-post-intervention comparisons, enabling the documentation of subtle changes in behaviours over time. The next aim of the project seeks to identify the impacts of the 6-month coaching intervention by comparing pre- and post-nutrition scores on the *CELEBRATE* scale. Future research could delve deeper into the impact of coaching interventions informed by detailed assessments on responsive feeding behaviours, providing a more nuanced understanding of the long-term effects.

In terms of practice, childcare providers, educators, consultants, dietitians, and public health interventionists stand to benefit from the insights provided by the *CELEBRATE* scale. The detailed evaluation of responsive feeding practices offers a targeted approach for interventions, allowing practitioners to focus on specific areas that may need improvement. The study underscores the importance of tailoring coaching interventions based on the unique needs and strengths identified in each childcare centre, emphasizing a personalized and context-specific approach to fostering responsive feeding environments and practices.

## Conclusion

Our study describes responsive feeding practices using our modified EPAO tool resulting in two separate scoring scales to advance the current assessment of responsive feeding in early learning and child care. In our study, educators in child care rooms of various sizes and child ages were engaging in beneficial practices to support children’s responsive feeding, especially in those behaviours highlighting what not to do, such as not using food to calm or encourage appropriate behaviour and not requiring children to stay seated at the table until finished eating all food. However, the scores also identified some responsive feeding practices requiring more support. It was important to observe and score these behaviours using our modified EPAO tool to allow for the measurement of current and relevant responsive feeding practices and environments, and to use the *CELEBRATE* scale to gain a complete description of the behaviours of multiple educators in the whole room, to enable more precise tracking of behaviour change over time, and to help inform areas of support needed for the coaching intervention. Adapting the EPAO tool for these uses is essential in gaining detailed, current, and tailored information to inform future relevant public health nutrition interventions through coaching in early learning and child care centres. The approach used in CELEBRATE Feeding embodies principles of responsive feeding to enable children’s self-regulation related to food to support their health and well-being, while also building the foundation for a positive relationship with food through enjoyable, pressure-free exposure and exploration.

## Supporting information

Campbell et al. supplementary material 1 Campbell et al. supplementary material

Campbell et al. supplementary material 2 Campbell et al. supplementary material

## Data Availability

The data for this study are available upon responsible request to the corresponding author and upon the signing of a data transfer agreement.

## Supplementary material

To view supplementary material for this article, please visit https://doi.org/10.1017/jns.2024.10

## References

[1] Miguel PM , Pereira LO , Silveira PP , et al. Early environmental influences on the development of children’s brain structure and function. Dev. Med. Child Neurol. 2019;61:1127–1133. 30740660 10.1111/dmcn.14182

[2] Tierney AL , Nelson CA. Brain development and the role of experience in the early years. Zero Three 2009;30:9–13. 23894221 PMC3722610

[3] Ardoin NM , Bowers AW. Early childhood environmental education: a systematic review of the research literature. Educ. Res. Rev. 2020;31:100353. 34173434 10.1016/j.edurev.2020.100353 PMC7348615

[4] Nelson CA , Scott RD , Bhutta ZA , et al. Adversity in childhood is linked to mental and physical health throughout life. BMJ 2020;371:m3048. 33115717 10.1136/bmj.m3048 PMC7592151

[5] Nurius PS , Green S , Logan-Greene P , et al. Life course pathways of adverse childhood experiences toward adult psychological well-being: a stress process analysis. Child Abuse Negl. 2015;45:143–153. 25846195 10.1016/j.chiabu.2015.03.008 PMC4470711

[6] Sinha M. Child care in Canada. 2014. Accessed January 2023. https://www150.statcan.gc.ca/n1/pub/89-652-x/89-652-x2014005-eng.htm

[7] Statistics Canada. The Daily — Survey on early learning and child care arrangements, 2022. 2022. Accessed July 2023. https://www150.statcan.gc.ca/n1/daily-quotidien/220601/dq220601a-eng.htm

[8] Archambault J , Côté D , Raynault M-F. Early childhood education and care access for children from disadvantaged backgrounds: using a framework to guide intervention. Early Child. Educ. J. 2020;48:345–352. 32226270 10.1007/s10643-019-01002-x PMC7089625

[9] Baker M , Gruber J , Milligan K. The long-run impacts of a universal child care program. Am. Econ. J. Econ. Policy 2019;11:1–26.

[10] Ulferts H , Wolf KM , Anders Y. Impact of process quality in early childhood education and care on academic outcomes: longitudinal meta-analysis. Child Dev. 2019;90:1474–1489. Wiley-Blackwell. 31407322 10.1111/cdev.13296

[11] Health Canada. Canada Health Act Annual Report 2011–2012. 2013. Accessed January 2024. https://www.canada.ca/en/health-canada/services/health-care-system/reports-publications/canada-health-act-annual-reports/report-2011-12.html

[12] Nishida C , Uauy R , Kumanyika S , et al. The joint WHO/FAO expert consultation on diet, nutrition and the prevention of chronic diseases: process, product and policy implications. Public Health Nutr. 2004;7:245–250. 14972063 10.1079/phn2003592

[13] Uauy R , Kain J , Mericq V , et al. Nutrition, child growth, and chronic disease prevention. Ann. Med. 2008;40:11–20. 18246473 10.1080/07853890701704683

[14] Sisson SB , Krampe M , Anundson K , et al. Obesity prevention and obesogenic behavior interventions in child care: a systematic review. Prev. Med. 2016;87:57–69. 26876631 10.1016/j.ypmed.2016.02.016

[15] Daniels LA. Feeding practices and parenting: a pathway to child health and family happiness. Ann. Nutr. Metab. 2019;74:29–42. 31234189 10.1159/000499145

[16] Haines J , Haycraft E , Lytle L , et al. Nurturing children’s healthy eating: position statement. Appetite 2019;137:124–133. 30797837 10.1016/j.appet.2019.02.007

[17] Health Canada. Do Canadian children meet their nutrient requirements through food intake alone? 2009. Accessed March 2019. http://publications.gc.ca/site/eng/356011/publication.html

[18] Scaglioni S , De Cosmi V , Ciappolino V , et al. Factors influencing children’s eating behaviours. Nutrients 2018;10:706. 29857549 10.3390/nu10060706 PMC6024598

[19] Black MM , Aboud FE. Responsive feeding is embedded in a theoretical framework of responsive parenting. J. Nutr. 2011;141:490–494. 21270366 10.3945/jn.110.129973 PMC3040905

[20] Finnane JM , Jansen E , Mallan KM , et al. Mealtime structure and responsive feeding practices are associated with less food fussiness and more food enjoyment in children. J. Nutr. Educ. Behav. 2017;49, 11–18.e1. Elsevier. 27707544 10.1016/j.jneb.2016.08.007

[21] Hurley KM , Cross MB , Hughes SO. A systematic review of responsive feeding and child obesity in high-income countries. J. Nutr. 2011;141:495–501. 21270360 10.3945/jn.110.130047 PMC3040906

[22] Shloim N , Edelson LR , Martin N , et al. Parenting styles, feeding styles, feeding practices, and weight status in 4–12 year-old children: a systematic review of the literature. Front. Psychol. 2015;6:1849. 10.3389/fpsyg.2015.01849 PMC4677105 26696920

[23] DiSantis K , Hodges E , Johnson S , et al. The role of responsive feeding in overweight during infancy and toddlerhood: a systematic review. Int. J. Obes. 2005 2011;35:480–492. 10.1038/ijo.2011.3 PMC6598438 21427696

[24] Dev DA , Garcia AS , Dzewaltowski DA , et al. Provider reported implementation of nutrition-related practices in childcare centers and family childcare homes in rural and urban Nebraska. Prev. Med. Rep. 2020;17:101021. Elsevier. 31908908 10.1016/j.pmedr.2019.101021 PMC6939097

[25] Health Canada. Healthy eating recommendations. *Can. Food Guide*. 2019. Accessed January 2023. https://food-guide.canada.ca/en/healthy-eating-recommendations/

[26] McIsaac J-LD , Richard B , Turner J , et al. Comparison of responsive feeding practices in child care and home environments in Nova Scotia. Can. J. Diet. Pract. Res. 2022;83:168–174. Dietitians of Canada. 36004728 10.3148/cjdpr-2022-017

[27] Ramsay SA , Branen LJ , Fletcher J , et al. ‘Are you done?’ Child care providers’ verbal communication at mealtimes that reinforce or hinder children’s internal cues of hunger and satiation. J. Nutr. Educ. Behav. 2010;42:265–270. 20579609 10.1016/j.jneb.2009.07.002

[28] Tovar A , Vaughn AE , Fallon M , et al. Providers’ response to child eating behaviors: a direct observation study. Appetite 2016;105:534–541. 27328098 10.1016/j.appet.2016.06.020 PMC5067159

[29] Dev DA , Speirs KE , Williams NA , et al. Providers perspectives on self-regulation impact their use of responsive feeding practices in child care. Appetite 2017;118:66–74. 28764901 10.1016/j.appet.2017.07.022

[30] Orrell-Valente JK , Hill LG , Brechwald WA , et al. “Just three more bites”: an observational analysis of parents’ socialization of children’s eating at mealtime. Appetite 2007;48:37–45. 17000028 10.1016/j.appet.2006.06.006 PMC2045650

[31] Redsell SA , Slater V , Rose J , et al. Barriers and enablers to caregivers’ responsive feeding behaviour: a systematic review to inform childhood obesity prevention. Obes. Rev. 2021;22:e13228. 33779040 10.1111/obr.13228

[32] Wood AC , Blissett JM , Brunstrom JM , et al. Caregiver influences on eating behaviors in young children. J. Am. Heart Assoc. 2020;9:e014520. American Heart Association. 32389066 10.1161/JAHA.119.014520 PMC7660848

[33] McIsaac J-LD , MacQuarrie M , Barich R , et al. Responsive feeding environments in childcare setting: a scoping review of the factors influencing implementation and sustainability. Int. J. Environ. Res. Public. Health 2022;19:11870. 36231167 10.3390/ijerph191911870 PMC9564844

[34] Shim JE , Kim J , Lee Y , et al. Fruit and vegetable intakes of preschool children are associated with feeding practices facilitating internalization of extrinsic motivation. J. Nutr. Educ. Behav. 2016;48:311–317.e1. 26925802 10.1016/j.jneb.2016.01.003

[35] Swindle T , Sigman-Grant M , Branen LJ , et al. About feeding children: factor structure and internal reliability of a survey to assess mealtime strategies and beliefs of early childhood education teachers. Int. J. Behav. Nutr. Phys. Act. 2018;15:85. 30200993 10.1186/s12966-018-0717-x PMC6131865

[36] Elford L , Brown A. Exploring child-feeding style in childcare settings: how might nursery practitioners affect child eating style and weight? Eat. Behav. 2014;15:314–317. 24854825 10.1016/j.eatbeh.2014.04.001

[37] Hughes SO , Patrick H , Power TG , et al. The impact of child care providers’ feeding on children’s food consumption. J. Dev. Behav. Pediatr. 2007;28:100. 17435460 10.1097/01.DBP.0000267561.34199.a9

[38] Ward D , Hales D , Haverly K , et al. An instrument to assess the obesogenic environment of child care centers. Am. J. Health Behav. 2008;32:380–386. 18092898 10.5555/ajhb.2008.32.4.380

[39] Children’s Healthy Weight Research Group. Resources | Environment and Policy Assessment and Observation (EPAO). 2018. Accessed July 2023. https://chwr.web.unc.edu/resources/

[40] Swinburn B , Egger G , Raza F. Dissecting obesogenic environments: the development and application of a framework for identifying and prioritizing environmental interventions for obesity. Prev. Med. 1999;29:563–570. 10600438 10.1006/pmed.1999.0585

[41] Dev DA , McBride BA , Speirs KE , et al. “Great job cleaning your plate today!” determinants of child-care providers’ use of controlling feeding practices: An exploratory examination. J. Acad. Nutr. Diet. 2016;116:1803–1809. 27650534 10.1016/j.jand.2016.07.016

[42] Chilman L , Kennedy-Behr A , Frakking T , et al. Picky eating in children: a scoping review to examine its intrinsic and extrinsic features and how they relate to identification. Int. J. Environ. Res. Public. Health 2021;18:9067. Multidisciplinary Digital Publishing Institute. 34501656 10.3390/ijerph18179067 PMC8431657

[43] Fox MK , Devaney B , Reidy K , et al. Relationship between portion size and energy intake among infants and toddlers: evidence of self-regulation. J. Am. Diet. Assoc. 2006;106:77–83. 10.1016/j.jada.2005.09.039 16376632

[44] Galloway A , Fiorito L , Francis L , et al. ‘Finish your soup’: counterproductive effects of pressuring children to eat on intake and affect. Appetite 2006;46:318–323. 16626838 10.1016/j.appet.2006.01.019 PMC2604806

[45] Michie S , van Stralen MM , West R. The behaviour change wheel: a new method for characterising and designing behaviour change interventions. Implement. Sci. 2011;6:42. 21513547 10.1186/1748-5908-6-42 PMC3096582

[46] Michie S , Atkins L , West R. The Behaviour Change Wheel: A Guide to Designing Interventions. 1st ed. Great Britain, UK: Silverback Publishing; 2014.

[47] Tarasuk V , Fafard St-Germain A-A , Mitchell A. Geographic and socio-demographic predictors of household food insecurity in Canada, 2011–12. BMC Public Health 2019;19: 1–12. 10.1186/s12889-018-6344-2 PMC6318847 30606152

[48] Newell FD , Williams PL , Watt CG. Is the minimum enough? Affordability of a nutritious diet for minimum wage earners in Nova Scotia (2002–2012). Can. J. Public Health Rev. Can. Sante Publique 2014;105:158–165. 10.17269/cjph.105.4322 PMC6972235 25165833

[49] Government of Canada SC. Obesity in Canadian Adults, 2016 and 2017. 2018. Accessed December 2023. https://www150.statcan.gc.ca/n1/pub/11-627-m/11-627-m2018033-eng.htm

[50] Tovar A , Vaughn AE , Fisher JO , et al. Modifying the Environment and Policy Assessment and Observation (EPAO) to better capture feeding practices of family childcare home providers. Public Health Nutr. 2019;22:223–234. Cambridge University Press. 30378521 10.1017/S1368980018002665 PMC6365011

[51] Vaughn AE , Mazzucca S , Burney R , et al. Assessment of nutrition and physical activity environments in family child care homes: modification and psychometric testing of the Environment and Policy Assessment and Observation. BMC Public Health 2017;17:1–11. 28851348 10.1186/s12889-017-4686-9 PMC5576128

[52] Ward DS , Mazzucca S , McWilliams C , et al. Use of the Environment and Policy Evaluation and Observation as a Self-Report Instrument (EPAO-SR) to measure nutrition and physical activity environments in child care settings: validity and reliability evidence. Int. J. Behav. Nutr. Phys. Act. 2015;12:124. 26410387 10.1186/s12966-015-0287-0 PMC4583722

[53] Byrne RA , Baxter K , Irvine S , et al. Feeding practices in Australian early childhood education and care settings. Public Health Nutr. 2022;25:303–311. 34558401 10.1017/S1368980021004055 PMC8883764

[54] Statistics Canada. Population centre and rural area classification 2016. 2017. Accessed March 2023. https://www.statcan.gc.ca/en/subjects/standard/pcrac/2016/introduction

[55] Fallon M , Halloran K , Gorman K , et al. Self-reported and observed feeding practices of Rhode Island Head Start teachers: Knowing what not to do. Appetite 2018;120:310–317. 28916196 10.1016/j.appet.2017.09.009

[56] Government of Nova Scotia. Manual for Food and Nutrition in Regulated Child Care Settings, 87; 2011.

[57] The P.E.I. Healthy Eating Alliance, The Prince Edward Island Department of Education and Early Childhood Development & the Children’s Secretariat Partnerships for Children. Healthy living guidelines for early learning and child care centres on Prince Edward Island. 2012. Accessed July 2023. http://www.gov.pe.ca/photos/original/eecd_healthyliv.pdf

[58] Ajzen I , Joyce N , Sheikh S , et al. Knowledge and the prediction of behavior: the role of information accuracy in the theory of planned behavior. Basic Appl. Soc. Psychol. 2011;33:101–117.

[59] Baumeister RF , Bratslavsky E , Finkenauer C , et al. Bad is stronger than good. Rev. Gen. Psychol. 2001;5:323–370. SAGE Publications Inc.

[60] Farrer Mackie J , Marshall J , Alkon A , et al. Mealtime best practices and infection control in early care and education centres during COVID-19. Child Care Health Dev. 2022;48:990–1000. 35102591 10.1111/cch.12979

[61] Farrer Mackie J , Marshall J , Gray HL , et al. “Just sit and eat.” Adult and child mealtime responsibilities in early care and education centers during COVID-19 in Florida. Ecol. Food Nutr. 2022;61:559–575. 35575781 10.1080/03670244.2022.2073352

[62] Lafave L , Webster AD , McConnell C. Impact of COVID-19 on early childhood educator’s perspectives and practices in nutrition and physical activity: a qualitative study. Early Child. Educ. J. 2021;49:935–945. 33935480 10.1007/s10643-021-01195-0 PMC8068455

