# Supplementary figures and images for: Advancing assessment of responsive feeding environments and practices in child care
Source: J Nutr Sci. 2024 Mar 7;13:e14. doi: 10.1017/jns.2024.10 (PMC10988165; doi:10.1017/jns.2024.10)

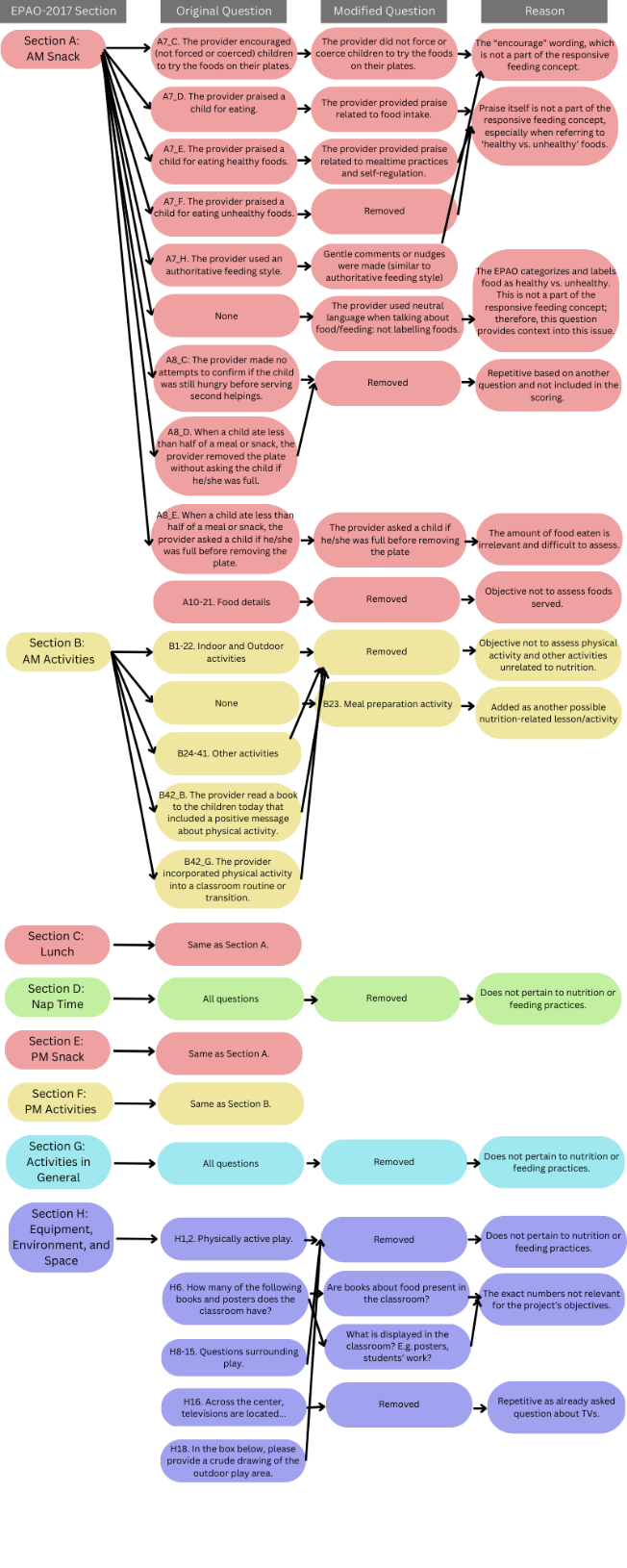


**Additional file 1**. Map of modifications made to EPAO-2017.

Supplement: Campbell et al. supplementary material 1 — Campbell et al. supplementary material [file S2048679024000107sup001.docx]
