# Supplementary material for: Advancing assessment of responsive feeding environments and practices in child care
Source: J Nutr Sci. 2024 Mar 7;13:e14. doi: 10.1017/jns.2024.10 (PMC10988165; doi:10.1017/jns.2024.10)

**Additional file 2.** Examples illustrating how the scoring in both our modified EPAO and the *CELEBRATE* scales can differ. A) Are examples of how the same score could arise for both scales given different scenarios and b) are examples of how the same situations would result in different scores on both scales.

a)


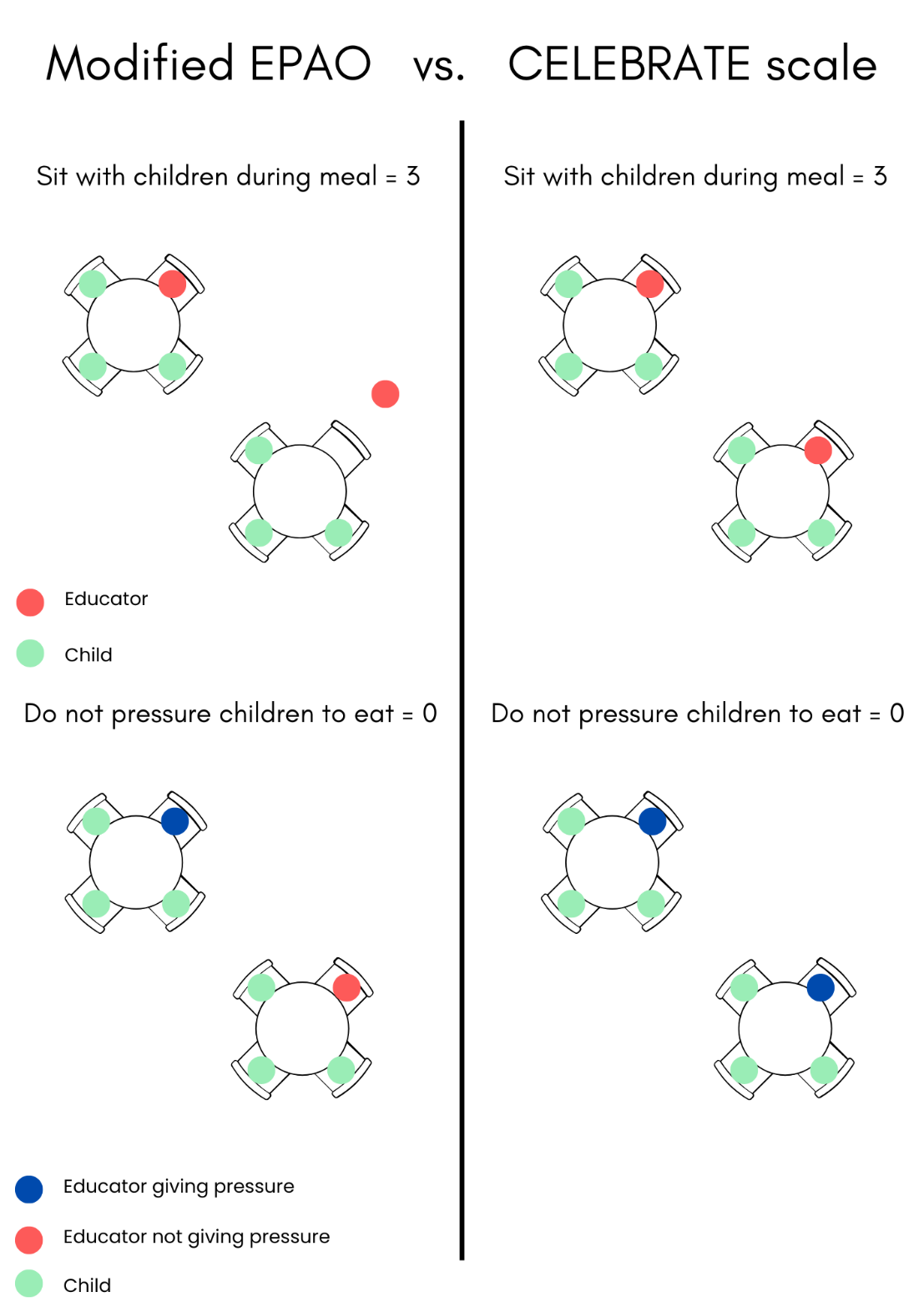


b)


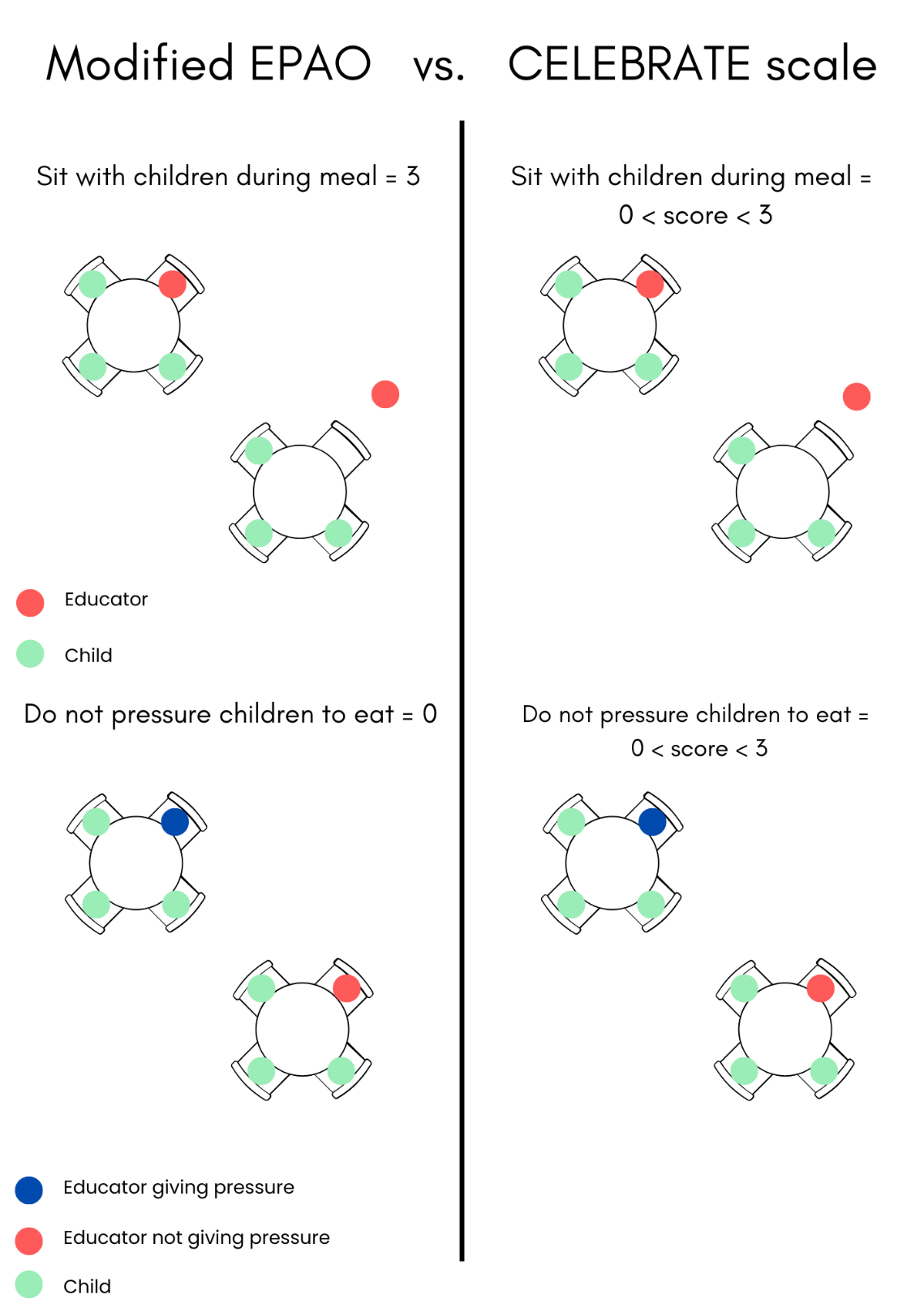

Supplement: Campbell et al. supplementary material 2 — Campbell et al. supplementary material [file S2048679024000107sup002.docx]
